# Antifungal De-Escalation Is Safe in Critically Ill Patients Treated For Suspected Or Documented Invasive Candidiasis. Data From The Amarcand2 Study

**DOI:** 10.1186/2197-425X-3-S1-A5

**Published:** 2015-10-01

**Authors:** S Bailly, O Leroy, P Montravers, JM Constantin, H Dupont, D Guillemot, O Lortholary, JP Mira, PF Perrigault, JP Gangneux, E Azoulay, JF Timsit

**Affiliations:** University of Grenoble, Grenoble, France; INSERM UMR 1137, IAME Team 5, Paris, France; CHU Grenoble, Grenoble, France; CHU Tourcoing, Tourcoing, France; CHU Bichat, Paris, France; CHU Clermont Ferrand, Clermont Ferrand, France; CHU Amiens, Amiens, France; Hopital Necker, Paris, France; CHU Cochin, Paris, France; CHU Montpellier, Montpellier, France; University of Rennes, Rennes, France; Saint-Louis Hospital, Paris, France; INSERM, Biostatistics and Clinical Epidemiology, UMR 1153, Paris, France; Université Paris Diderot, Paris, France

## Introduction

Systemic Antifungal therapy (SAT) of invasive candidiasis (IC) needs to be started immediately upon clinical suspicion. Controversies exist then about potential harms from antifungal de-escalation (DE).(1) in cases of documented IC, early DE to fluconazole is recommended by US guidelines but discouraged in EU guidelines. in non-documented IC, no data are available to guide SAT DE. These questions are key issues however as the relationship between antifungal use and *Candida* antifungal resistance has been repeatedly demonstrated.

## Objectives

To investigate whether DE within 5 days of SAT initiation is associated with an increase in 28-day death in SAT-treated non-neutropenic adult ICU patients.

## Methods

Patients were recruited from AmarCAND2, a multicenter prospective observational study conducted in French ICUs (835 patients; 84 ICUs). The study included 647 non-neutropenic adult ICU patients who received SAT for a documented or suspected invasive candidiasis and who were alive and in the ICU after 5 days of SAT initiation. The study considered two groups: SAT DE group (patients switched from initial SAT to fluconazole or to SAT stop within five days of SAT initiation) and SAT continuation group (patient without switch or stop of the initial SAT within five days of SAT initiation). The average causal SAT DE effect on 28-day death was performed by using a double robust Inverse Probability of Treatment Weight (IPTW) estimation for observational data.

## Results

**Figure 1:**
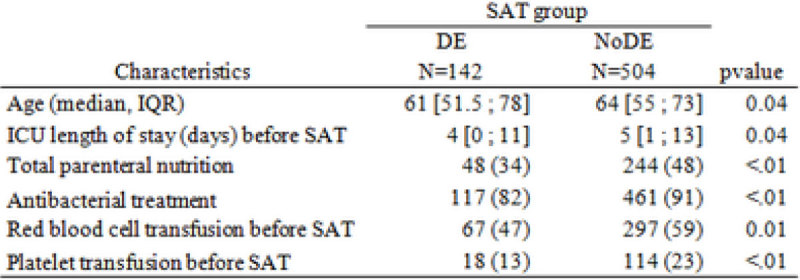
**Main baseline difference.**

Of 647 included patients, early DE at day 5 after SAT initiation was performed in 142 patients (22%) of whom 48 (34%) SAT was stopped before day 5. The initial SAT was mainly echinocandins (372 patients (58%)) and fluconazole (261 patients (40%)). 276 patients (43%) had a proven invasive candidiasis (candidemia: 41%, peritonitis: 47%, deep-seated invasive Candida infection: 18%; non-exclusive cases). Crude mortality before day 28 was 29% in the DE group, 26% in the no DE group. Main variables associated with DE are reported on the Table below. After adjustment on the baseline confounders, early SAT DE was not found to be associated with increased 28-day mortality (RR: 1.14, 95% CI [0.78-1.66]). Results are not different if analyses are restricted to proven IC (n = 276, RR = 0.89, 95% CI [0.53-1.49]), suspected IC (n = 371, RR = 0.99, 95% CI [0.58-1.68]), or first line candins therapy (n = 373, RR = 1.02, 95% CI [0.70-1.50]). DE increased the number of day alive without SAT (13 days [5-23] vs. 10 days [1-17] p < .01). Figure 1.

## Conclusions

In non-neutropenic adult critically ill patients with proven or suspected CI, SAT DE within 5 days was not found to be associated with increased day-28 mortality. Alternatively, SAT DE was found to be associated with decreased SAT consumption. These results show for the first time that SAT DE is safe, more particularly in cases of unproven invasive candidiasis.

## Grant Acknowledgment

MSD France

## References

[1] Deshpande (2013). IJAA.

